# Data in support of Gallium (Ga^3+^) antibacterial activities to counteract *E. coli* and *S. epidermidis* biofilm formation onto pro-osteointegrative titanium surfaces

**DOI:** 10.1016/j.dib.2016.01.024

**Published:** 2016-01-22

**Authors:** A. Cochis, B. Azzimonti, R. Sorrentino, C. Della Valle, E. De Giglio, N. Bloise, L. Visai, G. Bruni, S. Cometa, D. Pezzoli, G. Candiani, L. Rimondini, R. Chiesa

**Affiliations:** a Department of Health Sciences, Medical School, University of Piemonte Orientale (UPO), Via Solaroli 17, 28100 Novara, Italy; b Department of Biomedical, Surgical and Dental Sciences, Università di Milano/ University of Milan, via Beldiletto 1, 20142 Milan, Italy; c Consorzio Interuniversitario Nazionale per la Scienza e Tecnologia dei Materiali (INSTM), Via Giusti 9, Local Unit Piemonte Orientale, 50121 Firenze, Italy; d Department of Chemistry, Materials and Chemical Engineering ‘G.Natta’, Politecnico di Milano, Via Mancinelli 7, 20131 Milano, Italy; e Department of Chemistry, University of Bari “Aldo Moro”, Via E. Orabona 4, 70126 Bari, Italy; f Molecular Medicine Department, Center for Health Technologies (CHT), UdR INSTM, University of Pavia, Viale Taramelli 3/b, 27100 Pavia, Italy; g Department of Occupational Medicine, Toxicology and Environmental Risks, Laboratory of Nanotechnology, Salvatore Maugeri Foundation, IRCCS, Via Boezio 28, 27100 Pavia, Italy; h Department of Chemistry - Physical-Chemistry Section, University of Pavia, Viale Taramelli 12, 27100 Pavia, Italy; i Jaber Innovation s.r.l., Via Calcutta 8, 00100 Roma, Italy

**Keywords:** ASD, Anodic Spark Deposition, AgCis, Antibacterial agent (AgNO3) with chelating agent (L-Cysteine), AgNPs, Silver NanoParticles, GaCis, Antibacterial agent (Ga (NO3)3) with chelating agent (L-Cysteine), GaOss, Antibacterial agent (Ga (NO3)3) with chelating agent (oxalic acid), MTT, 3-[4,5-dimethylthiazol-2-yl]-2,5 diphenyl tetrazolium bromide, SEM, Scanning Electron Microscopy, *E. coli*, *S. epidermidis*, Biofilm, Gallium, Silver, Titanium, Anodic Spark Deposition

## Abstract

This paper contains original data supporting the antibacterial activities of Gallium (Ga^3+^)-doped pro-osteointegrative titanium alloys, obtained via Anodic Spark Deposition (ASD), as described in “The effect of silver or gallium doped titanium against the multidrug resistant *Acinetobacter baumannii*” (Cochis et al. 2016) [1].

In this article we included an indirect cytocompatibility evaluation towards Saos2 human osteoblasts and extended the microbial evaluation of the Ga^3+^ enriched titanium surfaces against the biofilm former *Escherichia coli* and *Staphylococcus epidermidis* strains. Cell viability was assayed by the Alamar Blue test, while bacterial viability was evaluated by the metabolic colorimetric 3-[4,5-dimethylthiazol-2-yl]-2,5 diphenyl tetrazolium bromide (MTT) assay. Finally biofilm morphology was analyzed by Scanning Electron Microscopy (SEM). Data regarding Ga^3+^ activity were compared to Silver.

**Specifications Table**

**Table:** 

Subject area	*Biomaterials*
More specific subject area	*Biomimetic pro-osteointegrative antibacterial materials*
Type of data	*Graphics (histograms), SEM images*
How data was acquired	*Viability via colorimetric assay evaluated by Optical density by spectrophotometer (Tecan Genius Plus; Tecan Italia, Cernusco Sul Naviglio, Italy); images by SEM (Zeiss EVO-MA10; Carl Zeiss, Oberkochen, Germany)*
Data format	*Analyzed: means ± standard deviations and statistics*
Experimental factors	*Grade II titanium disks were enriched with silicon, calcium, phosphorous and sodium using the Anodic Spark Deposition technique to obtain biomimetic surfaces. Silver and gallium were then added as antibacterial agents, using cysteine and oxalic acid as chelating agents*
Experimental features	*Cytocompatibility of specimens was evaluated towards Saos2 cells by Alamar blue assay until 21 days; silver and gallium coating bacteria killing activity was evaluated against 72hs mature E. coli and S. epidermidis biofilm by MTT assay. Images of the contaminated surfaces were obtained by SEM*
Data source location	*Saos2 human osteoblasts were purchased from European Collection of Cell Cultures (ECACC 89050205, distributed by Sigma-Aldrich, Milan, Italy); E. coli PHL628 and S. epidermidis RP62A isolates were kindly provided by the Universities of Pavia (Italy) and Dublin (Ireland), respectively*
Data accessibility	*Data are available in this article*

Value of the data

• Anodic Spark Deposition (ASD) is a novel suitable technique to apply biomimetic treatments to titanium surfaces.
• The data presented here provide new insights on Gallium and silver as useful antibacterial biomimetic treatments for pro-osteointegrative titanium based surfaces.

## 1 Data

The data here presented are directly available in this article and related to our previous publication by Cochis et al. [1]. We provide new data regarding the cytocompatibility (Supplementary Fig. 1) of electrochemically modified gallium or silver doped titanium alloys by Anodic Spark deposition and their antibacterial ability in counteracting biofilm formation produced by *Escherichia coli* and *Staphylococcus epidermidis* (Supplementary Figs. 2 and 3).

## 2 Experimental design, materials and methods

### 2.1 Specimen preparation

A biomimetic treatment, named SiB-Na, was made by the Anodic Spark Deposition (ASD) technique, as previously described [1]. The resulting SiB-Na specimens were then further modified by the addition of the antibacterial agents Gallium and Silver; obtained samples were identified as GaCis, GaOss, AgCis, and AgNPs respectively. For specimens preparation and characteristics please refer to Table 1 described in the Ref. [1]. Specimens were sterilized for 2 h by 70% ethanol immersion.

### 2.2 Eukaryotic cell cultivation

Human osteoblast Saos2 cells (purchased from the European Collection of Cell Cultures, ECACC number 89050205, provided by Sigma (Milan, Italy) were cultivated in McCoy medium (Sigma) supplemented with 15% fetal bovine serum (FBS, Sigma), 1% (v/v) Sodium Pyruvate (Sigma), 1% (v/v) and Penicillin-Streptomycin at 37 °C, 5% CO_2_ humid atmosphere. When cells reached 80–90% confluence, they were detached with trypsin-EDTA solution (0.25% in PBS, Sigma), collected and used for experiments.

### 2.3 Bacterial strains and culture conditions

The microorganisms used in this study, *E. coli* PHL628 [2] and *S. epidermidis* RP62A [3], are biofilm-producing strains. *E. coli* PHL628 was provided by Dr Roberta Migliavacca (University of Pavia, Italy) whereas *S. epidermidis* RP62A was a gift from Prof. Tim Foster (Department of Microbiology, Dublin University, Ireland). *E. coli* PHL628 and *S. epidermidis* RP62A were routinely grown overnight, respectively in Luria Bertani Broth (LB) and in tryptic soy broth (TSB) (Difco Laboratories Inc., Detroit, MI, USA), under aerobic conditions at 37 °C using a shaker incubator (New Brunswick Scientific Co., Edison, NJ, USA).

### 2.4 Indirect evaluation of cytocompatibility

Titanium disks were sterilized by 100% ethanol immersion (1 mL/specimen) followed by UV irradiation (235 nm; 30 min/side). After washing with PBS, they were submerged in 2 mL of complete McCoy cell culture medium (Sigma-Aldrich, Milan, Italy); 1% (v/v) sodium pyruvate (Sigma), 1% (v/v) Penicillin-Streptomycin solution (Sigma), and 15% (v/v) FBS (Fetal Bovine Serum, Sigma) were added to each well [4] and incubated for 21 days at 37 ± 2 °C in a H_2_O saturated atmosphere under constant shaking (50 rpm, using a VDRL DIGITAL MOD. 711/D shaker). After 1, 4, 7, 14 and 21 days, conditioned medium from each specimen was harvest and stored at −80 °C. Saos2 cells were seeded onto a 96-well plate at a density of 25×10^3^ cells/cm^2^ in 100 µL of complete McCoy cell culture medium. The cells were then incubated for 24 h at 37 °C, 95% relative humidity, and 5% v/v CO_2_. After 24 h the medium was replaced with 100 µL of cell medium conditioned for different times (5 wells/treatment) and with fresh medium (for control wells); plates were then incubated for 24 h, after which the medium was removed from each well and the plates were incubated for 3 h with 100 µL of Alamar solution. Cell viability was then measured using a spectrophotometer plate reader (Tecan Genius Plus; Tecan Italia, Cernusco Sul Naviglio, Italy) at excitation wavelength 540 nm and emission wavelength 590 nm.

### 2.5 Antibacterial activity

For biofilm growth, overnight cultures of *E. coli* PHL628 and *S. epidermidis* RP62A were diluted in their own culture medium containing 0.25% glucose [5]. Aliquots (1 mL) of the diluted bacterial suspensions were applied to the surfaces of 1 cm diameter disks, placed in 24-well tissue culture plates (Costar, Corning, NY, USA) and incubated for 72 hours at 37 °C. Biofilm that had formed on the disks was washed twice with PBS (137 mM NaCl, 2.7 mM KCl, 4.3 mM Na_2_HPO_4_, pH 7.4) to remove planktonic cells and loosely adhering bacteria [6]. The quantitative 3-[4,5-dimethylthiazol-2-yl]-2,5 diphenyl tetrazolium bromide (MTT Sigma Aldrich, St Louis, MO, USA) test was used to assess dehydrogenase activity, as an indicator of the metabolic state of the biofilm cells on the different surfaces [7]. Moreover, 72 h after culturing on the disk surface, the bacterial morphology was analyzed by SEM. Briefly, the disks were washed several times and then fixed for SEM with 2.5% glutaraldehyde in 0.1 M cacodylate buffer, pH 7.2, for 1 h at 4 °C. After additional washes, they were incubated with increasing concentrations of ethanol (25%, 50%, 75% and 96%) for 10 min each, dried to the critical point using an Emitech K-850 apparatus (Emitech, Ashford, Kent, UK), and placed on a mounting base. Finally, the disks were coated with gold and examined under a SEM (Zeiss EVO-MA10; Carl Zeiss, Oberkochen, Germany).

### 2.6 Statistical analysis of data

Data are expressed as means ± standard deviation. Statistical analysis was performed using the SPSS v20 software (IBM, Chicago, USA) setting the significance level at p<0.05. Data were compared by one-way ANOVA followed by Sheffè post-hoc analysis.

## Funding

B.A. was supported by “Fondazione Cariplo 2013”(prot. 2013–0954); A.C. and B.A. were supported by PRIN 2010–2011 (PRIN 20102ZLNJ5_006), financed by the Ministry of Education, University and Research (M.I.U.R.), Rome, Italy. L.V. would like to acknowledge financial support from MIUR, PRIN 2010–11 (prot. 2010FPTBSH_009). The providers of funds had no role in study design, data collection and analysis, decision to publish, or preparation of the manuscript.

## Appendix A Supplementary material

Supplementary material ***Supplementary*****Supplementary Figure 1**. Viability of human osteoblasts (Saos2) after 1-4-7-14 and 21 days in direct contact with medium conditioned by contact with test materials. No statistically significant differences were noted versus controls (Ti) (*p*>0.05), confirming that no toxic compounds were released. Bars represent means ± standard deviation.

Supplementary material **Supplementary Figure 2 A-B**. *in vitro* evaluation of antibacterial activity of treated and untreated titanium disks against *E. coli* PHL628 cells. Data are expressed as percentage of bacteria growth on treated versus untreated disks (the latter being set to 100%) (n=3) (A). SEM images of *E. coli* PHL628 biofilms on the differently treated titanium specimens are shown (B). Scale bar= 10 um, magnification 5.000x (insert: scale bar =5 um; magnification 10.000x).

Supplementary material **Supplementary Figure 3 A-B**. Evaluation of *in vitro* antibacterial activity of treated and untreated titanium disks against *S. epidermidis* RP62A cells. Data were expressed as the percentage of bacteria growth on treated versus untreated disks (the latter set to 100%) (n=3) (a). SEM images of *S. epidermidis* RP62A biofilms on the differently treated titanium disks are shown (b). Scale bar= 10 um, magnification 5,000x (insert: scale bar =5 um; magnification 10,000x).
