# Supplementary figures and images for: Data in support of Gallium (Ga3+) antibacterial activities to counteract E. coli and S. epidermidis biofilm formation onto pro-osteointegrative titanium surfaces
Source: Data Brief. 2016 Jan 22;6:758–62. doi: 10.1016/j.dib.2016.01.024 (PMC4744237; doi:10.1016/j.dib.2016.01.024)

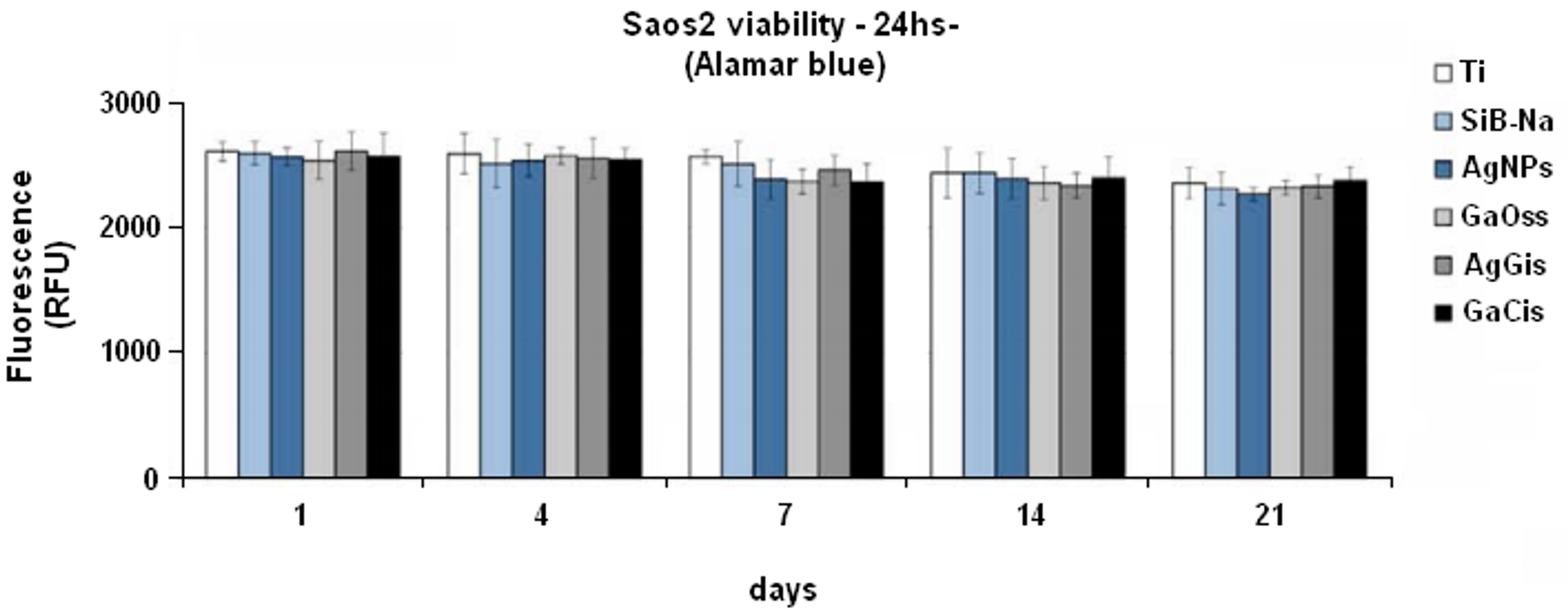

Supplement: Supplementary file 1 — Supplementary material SupplementarySupplementary Figure 1. Viability of human osteoblasts (Saos2) after 1-4-7-14 and 21 days in direct contact with medium conditioned by contact with test materials. No statistically significant differences were noted versus controls (Ti) (p>0.05), confirming that no toxic compounds were released. Bars represent means ± standard deviation. [file mmc1.zip › Supplementary Figure 1.tif]

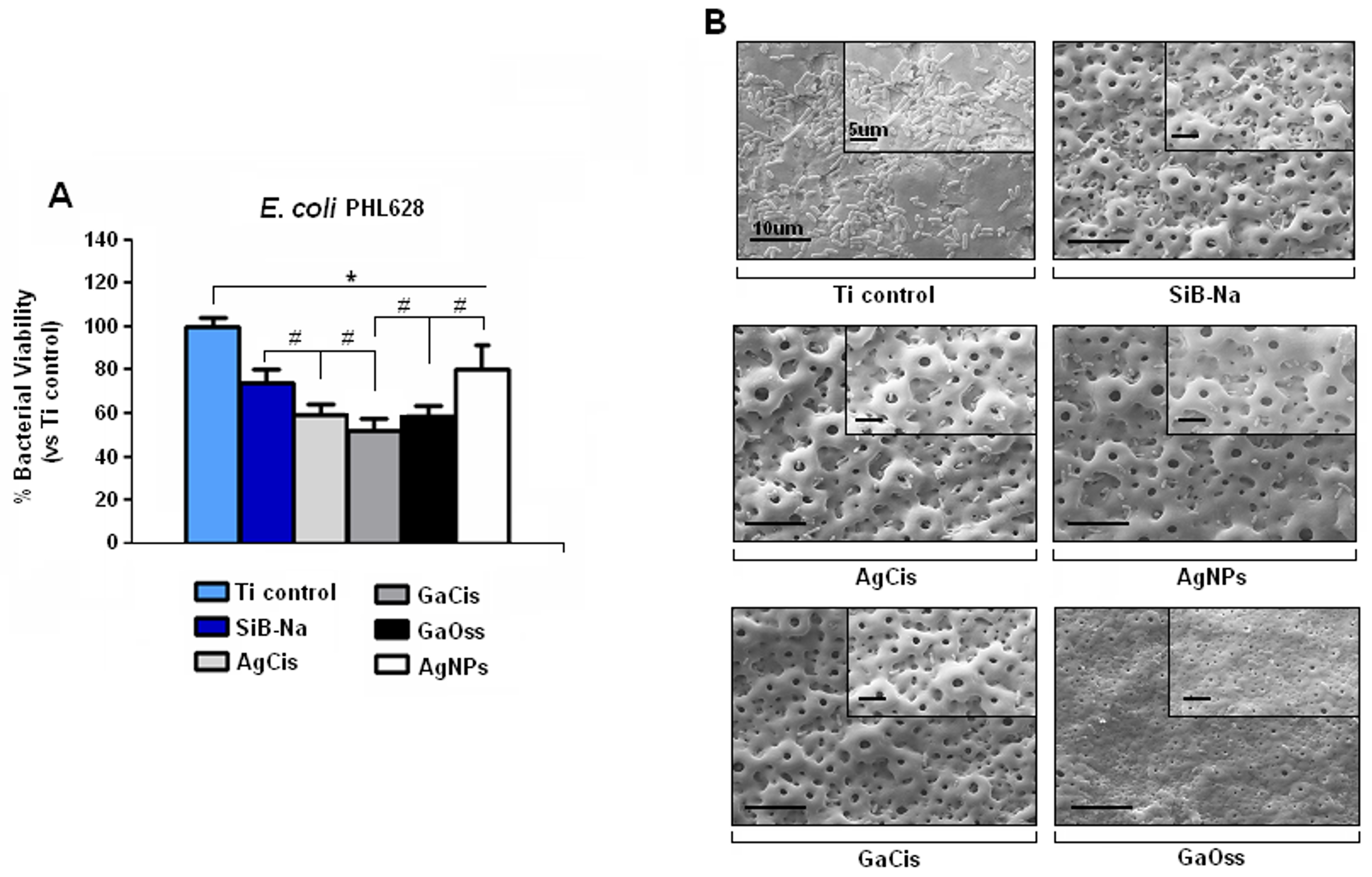

Supplement: Supplementary file 2 — Supplementary material Supplementary Figure 2 A-B. in vitro evaluation of antibacterial activity of treated and untreated titanium disks against E. coli PHL628 cells. Data are expressed as percentage of bacteria growth on treated versus untreated disks (the latter being set to 100%) (n=3) (A). SEM images of E. coli PHL628 biofilms on the differently treated titanium specimens are shown (B). Scale bar= 10 um, magnification 5.000x (insert: scale bar =5 um; magnification 10.000x). [file mmc2.zip › Supplementary Figure 2.tif]

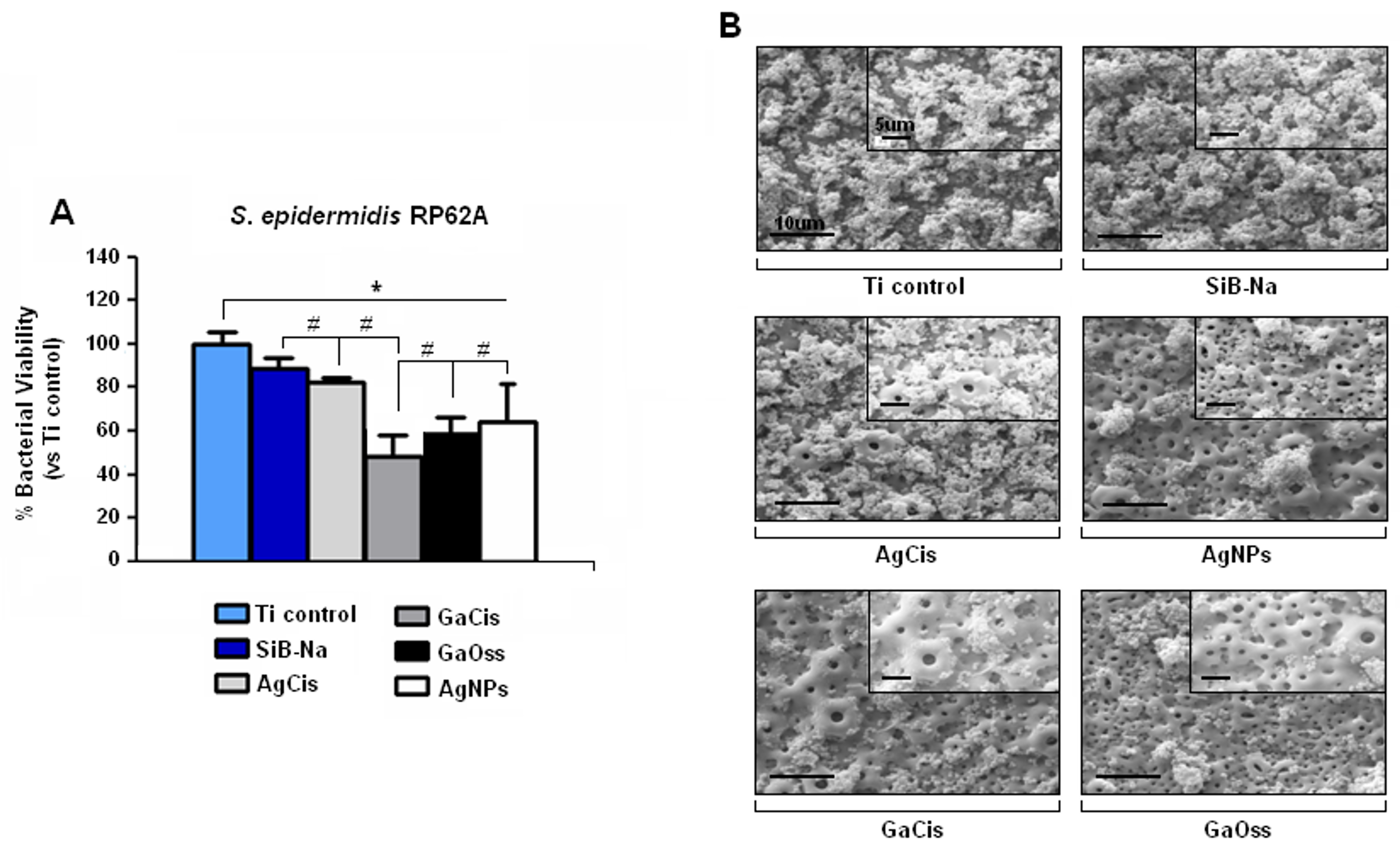

Supplement: Supplementary file 3 — Supplementary material Supplementary Figure 3 A-B. Evaluation of in vitro antibacterial activity of treated and untreated titanium disks against S. epidermidis RP62A cells. Data were expressed as the percentage of bacteria growth on treated versus untreated disks (the latter set to 100%) (n=3) (a). SEM images of S. epidermidis RP62A biofilms on the differently treated titanium disks are shown (b). Scale bar= 10 um, magnification 5,000x (insert: scale bar =5 um; magnification 10,000x). [file mmc3.zip › Supplementary Figure 3.tif]
